# A Hospital Dispute in Tasmania

**Published:** 1917-08-25

**Authors:** 


### The Hospital

The Workers' Newspaper of Administrative Medicine and Institutional Life, Administration, National Insurance and Health.

No. 1629, Vol. LXII. SATURDAY, AUGUST 25, 1917. PRICE ONE PENNY

### A HOSPITAL DISPUTE IN TASMANIA.

We have received somewhat late in the day, and probably in a very incomplete form, an account of a dispute which broke out early in the present year between the medical staffs of certain hospitals in Tasmania and the governing boards of these hospitals (see page 419). It is obvious that without full information we should not be justified in framing a final judgment on the points at issue, and we expressly exclude any pretence of our ability so to do. But the incident raises certain questions which are not without interest in themselves and which are related to principles that apply widely in the world of hospital management and control.

The main facts, so far as we can gather them, are anything but complicated, and the essence of the dispute is whether admission to the hospitals for purposes of treatment is to be determined by the honorary staff or by the committee of management. Not that this question is raised in an abstract form, for in such a discussion the verdict would manifestly be with the managing committee. The dispute is concerned with individual patients, or, rather, with an individual class of patients. This class is defined by the doctors as "rich and well-to-do," and the professional objection is to the admission of such patients to the hospital and the consequent pressure on the honorary staff to provide treatment for persons who are in a position to pay fees as private patients. Here, again, there would not seem to be grounds for quarrel, for, at all events in this country, no one would propose that rich or well-to-do persons should be able to claim professional services without the payment of fees. But in Tasmania the position is not quite so simple, as the hospitals are State-aided, and to them, therefore, each taxpayer makes a compulsory contribution. The advocates of a rigid equality thus find a "right" on the part of all members of the community to claim hospital treatment if they are inclined to this step. It would appear, too, that some persons from the class indicated have asserted this claim, and that the governing authorities have not felt themselves in a position to deny it. At all events, the doctors affirmed that patients other than the "poor" were admitted, and that unless this position was remedied they would retire from the hospital service. Further, we gather that the governing authorities did not dispute the contention of the staff, but pleaded that they had no power to refuse the admissions of which complaint was made. Regulations having the force of State laws barred the way, and under these the individual citizen, whatever his social rank or financial position, could demand for his personal benefit the advantages of the State-aided hospitals. Were this the complete statement of the position we should have no hesitation in expressing a complete sympathy with the doctors. It would be manifestly unjust that professional men giving honorary hospital service in the interests of the poor should be called on to treat gratuitously persons of the "rich and well-to-do" classes. If anyone who so desires may claim admission to hospital as a right due to him from the State, professional services performed in the hospital are services rendered to the State. It is by their agency that the State discharges its debt. In such circumstances the State manifestly must pay its agents, for, clearly, while the "poor" are, as they always have been, within the professional tradition of gratuitous treatment, this doctrine does not apply to the State. The State is not poor and needy. It does not ask for gratuitous service, and many will urge that it ought not to accept such service. Up to this point, therefore, the doctors, so far, at least, as their main contention is concerned, seem to have been in a strong position. They were fighting for a sound principle—namely, that while free professional advice and treatment may properly be given to the poor, no such obligation is due to a national or municipal organisation. In this latter case, doctors, like others who discharge public responsibilities, should be paid for their services. If the community in its corporate capacity undertakes obligations, all the citizens must, as taxpayers, take their share in discharging these obligations. As citizens, doctors come under this claim, just as they come under the moral claim to show kindliness and helpfulness to those who need succour. But no further obligation falls upon them, and if, to meet the common obligations incurred by the community, the services of doctors are necessary, those services must be paid for according to a reasonable and agreed rate.

With such information as we have, however, we are by no means sure that the Tasmanian doctors conducted their argument with discretion. They pressed their resignation on the governing committee, as, indeed, they had a perfect right to do. But they do not seem to have recognised that the committee was bound by public regulations having the force of law. In such a position the wise policy is surely to agitate for a revision of the regulations, and, only when this effort fails, to adopt the threat of such an extreme measure as resignation. Moreover, it would seem that an offer was made by the Premier of the Colony to obtain legislation which would ensure that persons admitted to the hospital and in a position to pay fees for maintenance and treatment should be compelled to do so. The reply of the doctors was that there should be an absolute exclusion of such patients. Admitting that there may be modifying circumstances outside our knowledge, this demand does seem to us to go beyond the sound professional principle stated above. What patients shall be admitted to a State-aided hospital must be decided by the State. The doctors may reasonably say that, for such of these as are not "poor" they must be paid fees, and the amount must be adjusted by arrangement and discussion. Here is everything which sound professional principle demands. But to claim a right of veto in the admission of patients to hospitals supported by public funds is a new proposition, and we are not surprised that it is strongly contested.

